# Language ability in preterm children is associated with arcuate fasciculi microstructure at term

**DOI:** 10.1002/hbm.23632

**Published:** 2017-05-04

**Authors:** Piergiorgio Salvan, J. Donald Tournier, Dafnis Batalle, Shona Falconer, Andrew Chew, Nigel Kennea, Paul Aljabar, Ghislaine Dehaene‐Lambertz, Tomoki Arichi, A. David Edwards, Serena J. Counsell

**Affiliations:** ^1^ Centre for the Developing Brain Division of Imaging Sciences & Biomedical Engineering, King's College London United Kingdom; ^2^ Neonatal unit, St. George's University Hospital NHS London United Kingdom; ^3^ INSERM‐CEA, Neurospin Center, Cognitive Neuroimaging Unit Gif‐Sur‐Yvette France; ^4^ Department of Bioengineering Imperial College London United Kingdom

**Keywords:** diffusion magnetic resonance imaging, infant, language development, brain, preterm birth

## Abstract

In the mature human brain, the arcuate fasciculus mediates verbal working memory, word learning, and sublexical speech repetition. However, its contribution to early language acquisition remains unclear. In this work, we aimed to evaluate the role of the direct segments of the arcuate fasciculi in the early acquisition of linguistic function. We imaged a cohort of 43 preterm born infants (median age at birth of 30 gestational weeks; median age at scan of 42 postmenstrual weeks) using high *b* value high‐angular resolution diffusion‐weighted neuroimaging and assessed their linguistic performance at 2 years of age. Using constrained spherical deconvolution tractography, we virtually dissected the arcuate fasciculi and measured fractional anisotropy (FA) as a metric of white matter development. We found that term equivalent FA of the left and right arcuate fasciculi was significantly associated with individual differences in linguistic and cognitive abilities in early childhood, independent of the degree of prematurity. These findings suggest that differences in arcuate fasciculi microstructure at the time of normal birth have a significant impact on language development and modulate the first stages of language learning. *Hum Brain Mapp 38:3836–3847, 2017*. © **2017 Wiley Periodicals, Inc.**

## INTRODUCTION

Comparative studies in humans and nonhuman primates have shown that the evolution of language has resulted from specific modifications of the cortical areas and pathways that mediate linguistic function [Rilling et al., 2008]. The arcuate fasciculus is a bilateral white‐matter fiber tract linking the posterior superior temporal cortex (Wernicke's area) to Brodmann area 44 in the frontal cortex (Broca's area) via a dorsal projection that arches around the Sylvian fissure [Catani et al., 2005; Rilling et al., 2008]. In the human brain, diffusion weighted imaging has shown that the organization and cortical terminations of this tract are strongly modified in comparison to primates and has demonstrated that the auditory regions of the temporal cortex have a higher probability of connection via the dorsal pathway with the frontal cortices [Rilling et al., 2008, 2011; de Schotten et al., 2012]. In contrast, axonal tracing studies in monkeys have shown that the arcuate fasciculus connects to more dorsally located regions, such as the extrastriate visual cortex [Petrides and Pandya, 1984; Schmahmann et al., 2007]. Taken together, these findings have generated the theory that the expanded direct dorsal pathway may be a key structure responsible for supporting the emergence of language in humans.

In human adults, the arcuate fasciculus has been proposed to play an important role in the core syntactic computation of complex sentences [Berwick et al., 2013], in verbal short‐term memory and in the perception of the phonetic structure of speech [Liberman and Mattingly, 1985]. It is also hypothesized to play a distinctive role in speech production via the integration of auditory and motor representation. Predominantly at the syllable level, it is thought to map sensory targets in the auditory cortex to motor programs coded in Broca's area [Hickok, 2012; Hickok and Poeppel, 2007]. Patients with injuries involving either the left or right arcuate fasciculus have impaired ability in phonological and word repetition tasks and in verbal short‐term memory [Alexander et al., 1987; Benson et al., 1973; Damasio and Damasio, 1980; Geschwind, 1965]. With regard to learning, the microstructural properties of the left direct segment of the arcuate fasciculus have been associated with the process of learning new words in adulthood [López‐Barroso et al., 2013], whilst improved performance in auditory verbal learning tasks are significantly associated with a less lateralized volumetric pattern of the direct pathways [Catani et al., 2007]. Children with Angelman Syndrome in whom neither the left nor right arcuate fasciculi can be identified on diffusion tractography have no oral language development, whereas when the left arcuate fasciculus cannot be identified language difficulties are always observed [Paldino et al., 2016; Wilson et al., 2011]. These observations confirm the crucial role of the arcuate fasciculus in speech acquisition [Berwick et al., 2013; Hickok and Poeppel, 2007].

As infants have impoverished language production and their reception abilities were thought to be limited to the supra‐segmental properties of speech, the role of the arcuate fasciculus has traditionally been considered to be secondary during the first stages of language acquisition. Although inferior frontal regions are activated in several fMRI studies in infants [Baldoli et al., 2015; Dehaene‐Lambertz et al., 2006; Perani et al., 2011; Shultz et al., 2014], the ventral pathway, comprising the uncinate and the inferior fronto‐occipital fasciculus, has been proposed to initially be the main functional linguistic pathway connecting temporal and frontal areas [Brauer et al., 2013; Dubois et al., 2015; Perani et al., 2011]. Recent advances in diffusion‐weighted imaging have enabled the investigation of white‐matter tracts thought to be involved in the acquisition of language and neurodevelopmental skills during the neonatal period [Brauer et al., 2013; Dubois et al., 2015; Perani et al., 2011]. These studies have suggested that, in contrast to the mature brain (where it terminates in Broca's area), the anterior direct segment of the arcuate fasciculus cannot be dissected after the premotor cortex [Dubois et al., 2015]. Whilst this finding may represent a genuine developmental difference in the extent of the arcuate fasciculus [Brauer et al., 2013; Dubois et al., 2015; Perani et al., 2011], it could also reflect the low angular resolution used in the diffusion weighted sequences, which may have limited delineation of the arcuate fasciculus in regions where the fibers cross with the cortico‐spinal tracts and the corpus callosum in the corona radiata [Dubois et al., 2015].

Premature birth is associated with verbal impairment, the severity of which increases with increasing prematurity at birth [Luu et al., 2009, 2011; van Noort‐van der Spek et al., 2012]. Previous studies of infant brain development have shown that white‐matter architecture is significantly altered following premature birth [Ball et al., 2014; Counsell et al., 2003; Hüppi et al., 1998; Rose et al., 2008] and the degree of this alteration is directly related to performance in specific neurodevelopmental domains [Bassi et al., 2008; Berman et al., 2009; Groppo et al., 2014]. It is thus possible that premature delivery also affects the white‐matter structures that subserve language function impacting on later linguistic behavior.

To address the question of whether or not the arcuate fasciculus is a specific neurolinguistic precursor in early human infancy; and to assess whether the degree of prematurity affects arcuate fasciculus microstructure and drives the relationship with later linguistic behavior, we used high *b* value high‐angular resolution diffusion‐weighted imaging (HARDI) in a cohort of 43 preterm born infants at term equivalent age and assessed their linguistic developmental performance at 2 years. We hypothesized that intersubject differences in composite linguistic skills at 2 years would be associated with term equivalent fractional anisotropy (FA) of the left and right arcuate fasciculi. To act as a control, we tested whether any relationship between brain structure and language performance could also be associated with FA values of the cortico‐spinal tracts and the superior longitudinal fasciculi.

## MATERIALS AND METHODS

### Infants

Preterm infants were recruited as part of the Evaluation of Preterm Imaging study (Eprime), and were imaged at term equivalent age over a 3 year period (2010–2013) at Queen Charlotte's and Chelsea Hospital, London. The study was reviewed and approved by the National Research Ethics Service, and all infants were studied following written consent from their parents. A cohort of 43 preterm born infants [median age at birth of 30.14 gestational (GA) weeks; range 24–32; 18 females] with no evidence of focal abnormality on MRI were imaged using high‐angular resolution diffusion‐weighted neuroimaging at 42.14 postmenstrual (PMA) weeks (range 39–46) (Table 1), and followed up to around 22 months of age to assess their neurodevelopmental performance.

**Table 1:** Infant characteristics

Characteristic	Value
Median (range) GA at birth (weeks)	30 (24 – 33)
Median (range) birth weight (grams)	1205 (645 – 1990)
Median (range) PMA at MRI (weeks)	42 (39 – 46)
Female, no (%)	18 (42%)
Chorioamnionitis, no (%)	1 (2%)
Intrauterine growth restriction, no (%)	7 (16%)
Median (range) mechanical ventilation (days)	0 (0 – 40)
Necrotizing enterocolitis requiring surgery, no (%)	1 (2%)
Mean (± SD) parental SES	17.4293 (±8.0772)

### Acquisition of MRI Imaging Data at Term Equivalent Age

All MRI studies were supervised by an experienced pediatrician or nurse trained in neonatal resuscitation. Pulse oximetry, temperature, and heart rate were monitored throughout the period of image acquisition; hearing protection in the form of silicone‐based putty placed in the external ear (President Putty, Coltene; Whaledent) and Mini‐muffs (Natus Medical) was used for each infant. Sedation (25–50 mg/kg oral chloral hydrate) was administered to 33 infants. Imaging was acquired using an eight‐channel phased array head coil on a 3‐Tesla Philips Achieva MRI Scanner (Best, The Netherlands) located on the Neonatal Intensive Care Unit. Whole‐brain diffusion‐weighted MRI data were acquired in 64 noncollinear directions with *b* value of 2500 s/mm^2^ and 4 images without diffusion weighting (isotropic voxel size of 2 mm; TE = 62 ms; TR = 9000 ms). High‐resolution anatomical images were acquired with pulse sequence parameters: T1 weighted 3D MPRAGE: TR = 17 ms, TE = 4.6 ms, flip angle 13°, slice thickness 0.8 mm, field‐of‐view 210 mm, matrix 256 × 256 (voxel size: 0.82 × 0.82 × 0.8 mm); and T2 weighted fast‐spin echo: TR = 8670 ms, TE = 160 ms, flip angle 90°, slice thickness 2 mm with 1 mm overlap, field‐of‐view 220 mm, matrix 256 × 256 (effective voxel size: 0.86 × 0.86 × 1 mm).

### Neurodevelopmental Assessment at 22 Months

Standardized neurodevelopmental assessment at a median age of 22 months (range: 21–24 months; median of 20 months corrected for prematurity) was carried out by an experienced pediatrician or developmental psychologist with the Bayley Scales of Infant and Toddler Development, Third Edition (BSID‐III) [Bayley, 2006].

### MRI Data Preprocessing

T2‐weighted brain volumes were bias corrected, brain extracted and tissue segmented into white matter, gray matter, deep gray matter structures, and cerebrospinal fluid using a neonatal specific segmentation tool [Makropoulos et al., 2014]. Diffusion MRI volumes were first visually inspected to detect and exclude data with motion artifact. All subjects included in the study had 5 or fewer volumes excluded due to head‐motion. B0 field inhomogeneities, eddy currents, and intervolume motion were corrected using topup and eddy tools in FSL5 [Andersson and Sotiropoulos, 2016; Andersson et al., 2003; Smith et al., 2004, 2015]. B1 field inhomogeneity was corrected using ITK‐N4 [Tustison et al., 2010]. All rigid registrations in native subject space were estimated using FSL boundary‐based registration optimized for neonatal tissue contrasts [Toulmin et al., 2015]; and nonlinear registrations to the T2‐weighted template were estimated using Advanced Normalization Tools [Avants et al., 2008]. All transformation pairs were calculated independently and combined into a single transform to reduce interpolation error.

### Tractography of the Arcuate Fasciculi

**Figure 1:**
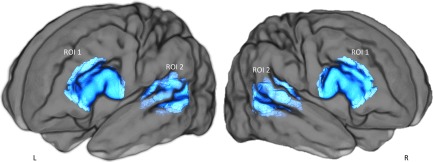
Regions of interest used to perform anatomically constrained spherical deconvolution tractography of the direct segment of the arcuate fasciculus. ROI 1: Broca's region (for the left and right hemisphere); ROI 2: Wernicke's region (for the left and right hemisphere). Fiber‐tracking of the arcuate fasciculus was performed in each subject's native space independently for both hemispheres. Only streamlines crossing both regions of interest were considered.

Estimation of fiber orientation distribution was computed through constrained spherical deconvolution [Tournier et al., 2004, 2007], with maximum spherical harmonic order of 8. We used the MRtrix3 package to perform anatomically constrained probabilistic tractography (http://www.mrtrix.org) [Smith et al., 2012; Tournier et al., 2012]. Fiber‐tracking of the arcuate fasciculus was performed in each subject's native space independently for both hemispheres, using a two‐region of interest approach. From the T2‐weighted template, we back‐projected two inclusion regions of interest and a seed‐plane. To maximize the chances of virtually dissecting the arcuate fasciculus, the seed‐plane was located in its direct dorsal pathway transverse to its antero‐posterior direction, and random seed‐streamlines were generated within it. The frontal region of interest was identified anterior to the central sulcus, to encompass the white matter of the posterior region of the inferior and middle frontal gyri. The temporal region of interest was defined in the white matter of the posterior part of the superior and middle temporal gyri (Fig. 1) [Forkel et al., 2014]. We extracted the median FA value along the reconstructed tract in both hemispheres as a measure of the white‐matter microstructure [Beaulieu, 2002; López‐Barroso et al., 2013].

### Tractography of the Cortico‐Spinal Tracts

To act as control regions, the left and right cortico‐spinal tracts were also delineated. From the T2‐weighted template, we back‐projected a seed‐region (an axial delineation of the pons) and two inclusion regions (the posterior limbs of the internal capsule and two axial‐planes at the level of the primary motor and somatosensory cortices). We then extracted the median FA value along the reconstructed tract in both hemispheres.

### Tractography of the Superior Longitudinal Fasciculi

To reconstruct the superior longitudinal fasciculi bilaterally we back‐projected a seed‐region (an axial plane delineating the parietal cortex) and an inclusion region (a coronal plane delineating the white matter area anterior of the motor cortex) [de Schotten et al., 2011]. We then extracted the median FA value along the reconstructed tract in both hemispheres.

## STATISTICAL ANALYSIS

To address the microstructural effect of neonatal development and early environmental factors linked to premature delivery, we used a general linear model (GLM) to test the linear association between arcuate fasciculi FA, cortico‐spinal tracts FA, and global white‐matter median FA, with (1) PMA at scan (covaried for GA at birth); and (2) GA at birth (covaried for PMA at scan). The number of days of ex‐utero life was highly correlated with PMA at scan and GA at birth (Pearson's correlation coefficient of postnatal age respectively with PMA and GA: *r* = 0.79; *P* < 10^−5^; *r* = −0.90; *P* < 10^−5^) and so we did not include this as a covariate in the model.

The primary goal of this study was to assess the role of the direct segments of the arcuate fasciculi in the early acquisition of linguistic function. To do this, we tested whether intersubject differences in composite linguistic skills at two years were associated with term equivalent FA of the left and right direct segments of the arcuate fasciculi.

We removed the effect of confound variables prior to the analysis; these were PMA (for the FA measures) and socioeconomic score (for linguistic skill measures). All features were standardized in the training sets to have zero mean and unit variance and the same transformation was then applied to the testing sets.

To test whether FA values of the left and right arcuate fasciculus was associated with composite linguistic skills at 2 years we first used cross‐validated Ordinary Least Squares (OLS) regression. However, coefficient estimates for OLS rely on the independence of the model terms and in this case the measured FA in left and right arcuate fasciculus were highly correlated (*r* = 0.58; *P* < 10^−5^). To overcome this, we used cross‐validated Ridge regression (a linear least squares variant with *L*_2_ regularization) and Partial Least Squares (PLS) regression. PLS is particularly suitable in cases where predictors are highly correlated or even collinear, that is, where standard regression is not appropriate [Hotelling, 1936; Wegelin, 2000]. The approach identifies linear combinations of the independent variables that optimally predict corresponding combinations of the dependent variables [Rosipal and Krämer, 2006]. Here, it was applied with mode A and deflation mode canonical [Wegelin, 2000].

We used leave‐one‐out cross‐validated PLS to assess whether intersubject differences in linguistic abilities at 2 years of age were associated with term equivalent FA of the left and right arcuate fasciculi. At each training iteration, the data for *n* − 1 subjects were used to train a PLS model; the learnt link was then used to generate the linguistic score for the left‐out subject. Following all iterations, the correlation between PLS FA scores and PLS language scores was assessed. We then extracted the PLS relative loadings of involvement averaged across all cross‐validation folds; the mean and standard deviation (SD) of these parameters were extracted to assess model stability.

As a control, we also used the same cross‐validated pipeline to test whether individual‐differences in linguistic performance were associated with left and right cortico‐spinal tracts FA, and left and right superior longitudinal fasciculus FA.

To test whether early environmental influences associated with preterm birth or global white‐matter volume [Northam et al., 2012] were driving the identified brain‐behavior link in a dose‐dependent fashion, we calculated the partial correlation between PLS FA scores and PLS language scores adjusting for GA at birth, global white matter volume, and sex.

Statistical significance was determined with nonparametric permutation testing (10,000 permutations) with correction for the Family wise error (FWE) rate [Winkler et al., 2014]. All analysis were performed using MATLAB (R2015b, The MathWorks, Natick, MA) and Scikit‐learn [Pedregosa et al., 2011].

## RESULTS

### Neurodevelopmental Assessment

At 2 years of age, the mean scores of the BSID‐III composite language and cognitive abilities were respectively 90 (SD ± 16.20) and 92 (SD ± 11.85), with a correlation between the two of *r* = 0.79; *P* = 10^−5^. No significant correlation was found between PMA at scan and composite language score. A trend toward significance was found between GA at birth and composite language score (*r* = 0.21; *P* = 0.09); a significant correlation was found between socioeconomic score (measured as the English Index of Multiple Deprivation) and composite language score (*r* = −0.28; *P* = 0.03).

### Impact of Prematurity on the Arcuate Fasciculus Microstructure

During the term equivalent period, prominent development occurred in the left and right arcuate fasciculi microstructure (respectively, FWE corrected *P*‐values = 0.0002 and 0.0013); in the cortico‐spinal tracts (respectively left and right, FWE corrected *P*‐values = 0. 0001 and 0.0016); and in the superior longitudinal fasciculus (respectively left and right, FWE corrected *P*‐values = 0. 0007 and 0.0014). Increased prematurity at birth was associated with significantly lower term equivalent FA of the left arcuate fasciculus (FWE corrected *P*‐value = 0.0130) and a trend toward lower FA in right arcuate fasciculus (FWE corrected *P*‐values = 0.0612; Table 2). We also found no significant difference between the left and right arcuate fasciculi in terms of term equivalent FA (Wilcoxon signed rank test: Zval = 0.07; *P* = 0.94) or tract length (corrected for brain volume; Wilcoxon signed rank test: Zval = 0.01; *P* = 0.99).

**Table 2:** The impact of degree of prematurity on white matter microstructure

	PMA (cov GA)	GA (cov PMA)
Left arcuate FA	0.0002	0.0130
Right arcuate FA	0.0013	0.0612
Left cortico‐spinal FA	0.0001	0.3448
Right cortico‐spinal FA	0.0016	0.8370
Left superior longitudinal FA	0.0007	0.9995
Right superior longitudinal FA	0.0004	0.5590

We assessed the effect of age at scan and gestational age at birth on arcuate fasciculi, cortico‐spinal tracts, and superior longitudinal fasciculi FA. Showing FWE corrected *P*‐values from GLM testing (10,000 permutations). A: Between 39 and 46 postmenstrual weeks, significant development (measured by PMA at scan covaried GA at birth) occurs in the arcuate fasciculi, cortico‐spinal tract, and superior longitudinal fasciculi microstructure. B: Increased prematurity at birth (measured by GA at birth covaried PMA at scan) is significantly associated with lower term equivalent FA of left arcuate fasciculus and a nonsignificant trend is seen in the right arcuate fasciculus.

### Term Equivalent Arcuate Fasciculus Microstructure Is Associated with Intersubject Differences in Linguistic Skills

**Figure 2:**
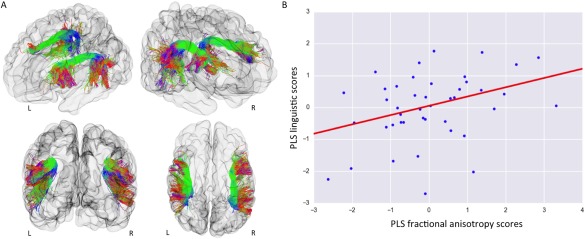
Intersubject differences in linguistic performance at two years were associated with term equivalent FA of the left and right arcuate fasciculus independently of degree of prematurity. A: Visualization of an infant brain and the reconstructed arcuate fasciculi from left‐frontal; right‐frontal; frontal and top view. The tracts are colored by direction: green for anterior‐posterior; red for left‐right; blue for superior‐inferior. B: Using cross‐validated partial‐least‐square regression, one statistically significant mode of brain‐behavior covariation between PLS FA scores and PLS language scores was identified (*r* = 0.36; FWE–corrected *P*‐value = 0.0110). Term equivalent FA of the left and right arcuate fasciculi was associated with individual differences in composite linguistic skills in early childhood. This link was still present even when controlling for degree of premature delivery measured by GA at birth (*r* = 0.32, FWE–corrected *P*‐value = 0.0230).

Although all cross‐validated regression analyses identified statistically significant brain‐behavior associations, the PLS regression model achieved greater nonparametric statistical significance when compared with OLS and Ridge regression (Table 3). The cross‐validated PLS analysis highlighted a statistically significant association between PLS FA scores and PLS language scores (*r* = 0.36; FWE–corrected *P*‐value = 0.0110; Fig. 2). Across folds, the PLS mode accounted for 72% of variance in the arcuate fasciculi FA. The mean of the identified PLS loadings (0.6650 for left and 0.7736 for right) was two orders of magnitude higher than their SD (0.0059 and 0.0099), highlighting strong model stability. The overall strong positive PLS loadings indicates that children who developed higher linguistic performance at two years were those with higher FA along both the left and right arcuate fasciculi at term equivalent age.

**Table 3:** Relationship between linguistic skills at 2 years and FA of the left and right arcuate fasciculi at term equivalent

	Arcuate fasciculi FA		Cortico‐spinal tracts FA		Superior Longitudinal fasciculi FA	
	*r*	FWE‐corrected *P*‐value	*r*	FWE‐corrected *P*‐value	*r*	FWE‐corrected *P*‐value
OLS	0.31	0.0275	−0.06	0.4839	−0.02	0.4325
Ridge	0.29	0.0305	−0.04	0.4556	−0.01	0.4166
PLS	0.36	0.0110	0.11	0.2785	0.16	0.2697

Showing rho correlation coefficient between FA at term equivalent and language scores at two years across different regression models and respective FWE‐corrected *P*‐values. The cross‐validated PLS regression demonstrated greater nonparametric statistical significance values. However, no statistically significant association was found when testing the link between linguistic scores and FA of the cortico‐spinal tracts or FA of the superior longitudinal fasciculus

The identified relationship remained significant when tested using partial correlation while adjusting for gestational age at birth, sex, and global white matter volume (*r* = 0.32, FWE–corrected *P*‐value = 0.0230). We then quantified the independent contribution of each variable in the relationship with language abilities (Table 5). This analysis confirmed that the only significant contribution was arcuate fasciculi FA (FWE‐corrected *P*‐value = 0.0226).

**Table 4:** PLS loadings in the initial sets of variables

				PLS loading
X space:				
	FA left arcuate fasciculus			0.66 ± SD 0.006
	FA right arcuate fasciculus			0.78 ± SD 0.009
Y space:				
	Linguistic skills			0.71 ± SD 0.003
	Cognitive skills			0.71 ± SD 0.002

Showing the PLS loadings of involvement in the identified link between the left and right arcuate fasciculus microstructure, and linguistic and cognitive performance. Mean PLS loadings of involvement ± SD averaged across folds. The PLS mode accounted for 72 and 71% of variance, respectively, in X and Y space. The small SDs of the estimated PLS loadings highlight strong model stability

To determine whether efficient linguistic abilities at 2 years were related to higher FA in both arcuate fasciculi, we tested the alternative hypothesis that lateralization in the arcuate fasciculi FA would be associated with later linguistic abilities. We found no significant association between the degree of asymmetry in the arcuate fasciculus microstructure [(Left FA − Right FA)/(Left FA + Right FA)] and composite linguistic skills at 2 years (GLM testing with 10,000 permutations: positive contrast FWE‐corrected *P*‐value = 0.8918; negative contrast FWE‐corrected *P*‐value = 0.1129).

**Figure 3:**
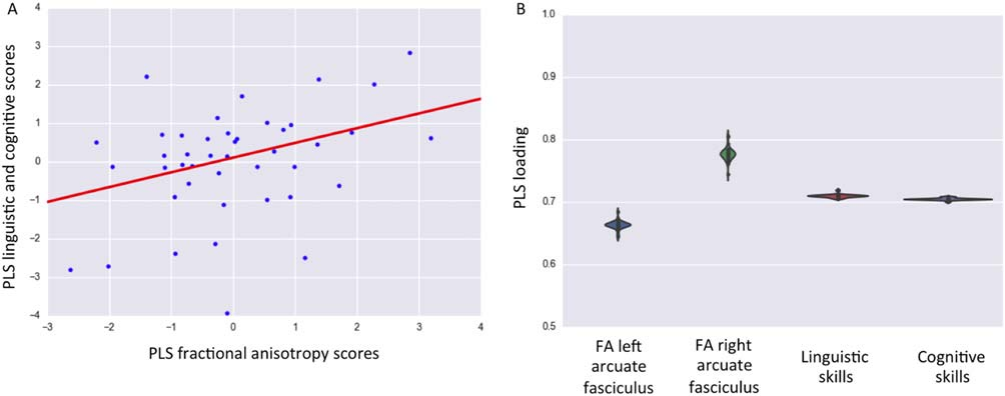
Association with inter‐subject differences in linguistic and cognitive performance at two years of age. A: A significant association was identified between PLS FA scores and PLS language and cognitive scores (*r* = 0.37; FWE–corrected *P*‐value = 0.0148). B: PLS loadings of involvement with respect to the initial X space (left and right arcuate fasciculi) and Y space (linguistic and cognitive skills). Note the small dispersion around the means highlight strong model stability. Higher composite linguistic and cognitive skills in early childhood were linked to higher FA of the left and right arcuate fasciculi at term equivalent.

When cognitive scores at 2 years were added to the model as an additional response variable, intersubject differences in linguistic and cognitive abilities remained associated with term‐equivalent FA of left and right arcuate fasciculus (*r* = 0.37; FWE–corrected *P*‐value = 0.0148). Higher linguistic and cognitive performance at two years of age were linked with higher FA along both the left and right arcuate fasciculi at term equivalent age. Across folds, the PLS mode accounted for 72 and 71% of variance respectively in X and Y space. High model stability in PLS loadings was highlighted also in this case (Fig. 3, Table 4).

**Table 5:** Independent contribution of each variable in the identified relationship with language abilities

Variables	FWE‐corrected *P*‐value
PLS FA scores arcuate fasciculus	0.0226
Gestational age	0.3712
Sex	0.1174
White‐matter volume	0.2694

The relation between arcuate fasciculus FA and linguistic skills at two years remained significant after correction for gestational age at birth, sex, and global white matter volume (partial correlation *r* = 0.32, FWE–corrected *P*‐value = 0.0230). Here, we used a GLM to assess the independent contribution of each predictor in the relationship with linguistic skills. Only the arcuate fasciculus FA was significantly associated with later language abilities, suggesting that the identified relationship was not driven by confounds of interest.

### Intersubject Differences in Linguistic Skills Are Not Associated with FA of the Cortico‐Spinal Tract or Superior Longitudinal Fasciculus at Term Equivalent Age

To test whether the identified relationship between term‐equivalent microstructure and linguistic abilities at two years of age was specific for arcuate fasciculus FA, we also tested the relationship with two other major white matter pathways: the cortico‐spinal tracts (which are known to be involved in motor function) and the superior longitudinal fasciculi, (a cortico‐cortical tract known to be involved in visual‐spatial attention and visual‐spatial working memory [de Schotten et al., 2011; Vestergaard et al., 2011].

**Figure 4:**
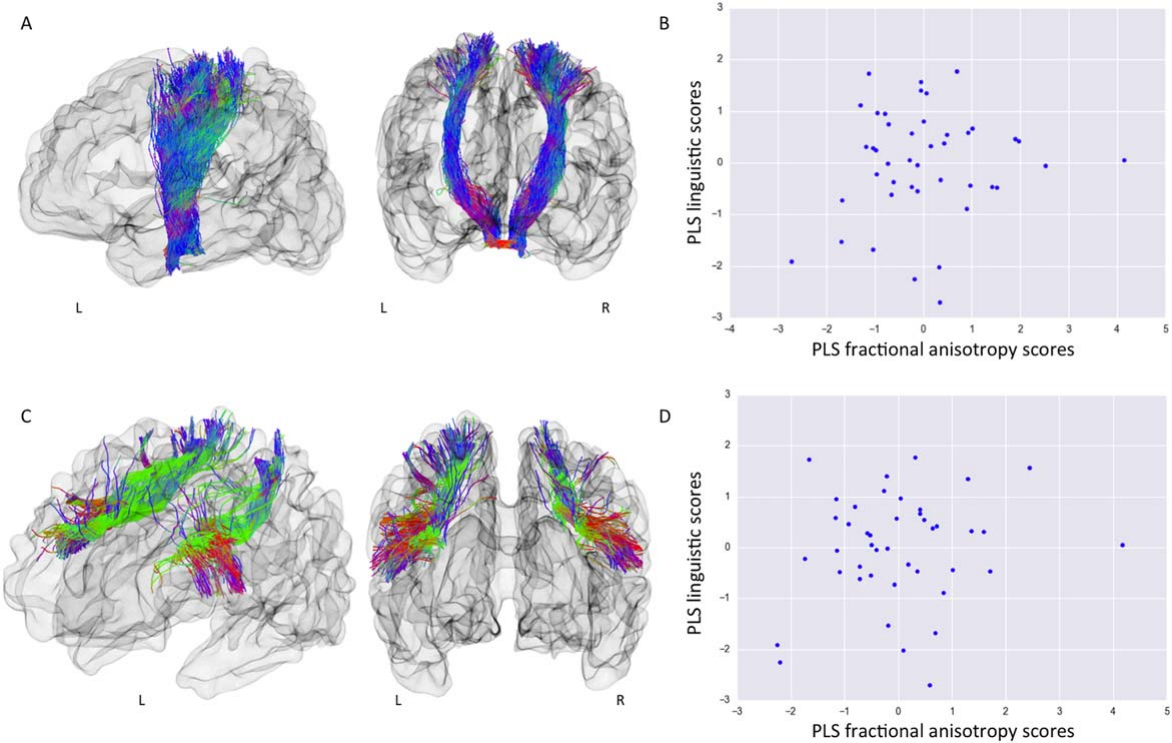
Term equivalent FA of the cortico‐spinal tracts and the superior longitudinal fasciculus is not associated with linguistic abilities at two years. A: Visualization of an infant brain and the reconstructed cortico‐spinal tracts from left and frontal view. B: Scatter plot of PLS cortico‐spinal tract FA scores versus PLS linguistic scores. Term‐equivalent FA of left and right cortico‐spinal tracts was not associated with linguistic skills at two years (*r* = 0.11, FWE–corrected *P*‐value = 0.2785), or with cognitive scores (*r* = 0.12, FWE–corrected *P*‐value = 0.3339). C: Visualization of an infant brain and the reconstructed superior longitudinal fasciculus from left‐frontal and frontal view. D: Scatter plot of PLS superior longitudinal fasciculus FA scores versus PLS linguistic scores. Term‐equivalent FA of left and right superior longitudinal fasciculus was not associated with linguistic skills at two years (*r* = 0.16, FWE–corrected *P*‐value = 0.2697). Of further interest, superior longitudinal fasciculus FA was significantly associated with cognitive scores (*r* = 0.32, FWE–corrected *P*‐value = 0.0390).

Linguistic skills at two years of age were not significantly associated with term‐equivalent FA within either the cortico‐spinal tracts (*r* = 0.11, FWE–corrected *P*‐value = 0.2785; Fig. 4) or the superior longitudinal fasciculi (*r* = 0.16, FWE–corrected *P*‐value = 0.2697). Of interest, FA within the superior longitudinal fasciculi was significantly associated with later general cognitive abilities (*r* = 0.32, FWE–corrected *P*‐value = 0.0390). There was no significant relationship between FA of the cortico‐spinal tracts and later cognition; *r* = 0.12, FWE–corrected *P*‐value = 0.3339).

## DISCUSSION

Infants demonstrate linguistic abilities at birth, including discriminating close phonemes [Dehaene‐Lambertz and Pena, 2001] and sentences from different languages [Mehler et al., 2002]. During the first year of postnatal life, rapid learning of the native language is evident and the discovery that the combinatorial properties of language can communicate information represent one of the most remarkable achievements of human learning. Although the underlying neural architecture of language acquisition is believed to be a distinct piece of the biological makeup of the human brain [Jackendoff and Pinker, 2005; Pinker, 1995], the precise neural mechanisms that allow human infants to develop this high‐order cognitive function remain unclear [Dehaene‐Lambertz and Spelke, 2015; Kuhl, 2010; Skeide and Friederici, 2016]. Preterm born children have impaired linguistic ability when compared with their term‐born peers, even in the absence of major disabilities, which can persist as a long‐lasting linguistic delay throughout childhood [van Noort‐van der Spek et al., 2012]. Studying this population therefore provides an opportunity to test hypotheses concerning infant brain mechanisms linked to language acquisition and to assess the environmental effects of early life exposure.

Noninvasive brain imaging techniques provide an opportunity to assess the neuroanatomical basis of early language acquisition. Diffusion‐weighted brain imaging allows the study of white‐matter FA, a measure sensitive to the underlying tissue microstructure [Beaulieu, 2002]. In adulthood, the brain architecture which sub‐serves language function is relatively well‐known [Berwick et al., 2013; Hickok and Poeppel, 2007; Price, 2012], with the arcuate fasciculus [Catani et al., 2005] representing a potential evolutionary marker of human linguistic capability.

This study shows that in preterm infants at the time of normal birth, well before the formal emergence of natural language, the microstructural properties of both the left and right arcuate fasciculi are associated with later linguistic abilities. Previous studies in adulthood and adolescence have linked its microstructural properties to word learning [López‐Barroso et al., 2013]; the development of reading skills [Yeatman et al., 2011, 2012]; sentence comprehension performance [Skeide et al., 2015], and it has been shown to support syntactic processing of language [den Ouden et al., 2012]. We found no significant difference in term equivalent FA and tract‐length between the left and right arcuate fasciculi. We also found that symmetry in the left and right arcuate fasciculi FA, rather than asymmetry, was linked to later efficient linguistic abilities. This is in accordance with the language deficiencies reported after both left and right hemispheric lesions in infants [Bates and Roe, 2001] and with the observation that arcuate fasciculus volumetric symmetry is linked to efficient auditory verbal learning in adulthood [Catani et al., 2007]. Previous studies in post‐term infants have shown a left hemispheric lateralization for speech processing in the posterior part of the superior temporal region [Baldoli et al., 2015; Dehaene‐Lambertz et al., 2002, 2006, 2010], but not for inferior frontal regions [Baldoli et al., 2015; Dehaene‐Lambertz et al., 2006]. Indeed, left‐lateralization in temporal areas increases during the first months of life [Baldoli et al., 2015; Perani et al., 2011; Shultz et al., 2014]. This bilateral linkage may also be due to the involvement of right frontal regions, which are also activated when infants listen to speech [Dehaene‐Lambertz et al., 2002, 2010], and are involved in attention, stimulus selection, and response to novelty. These are important processes for infants to comprehend social world requests, to communicate wants and needs, and to produce combinatorial‐grammatical sentences by the age of two years.

We may speculate on the role of the arcuate fasciculi during the first stages of language acquisition. It provides a direct link between speech production and perception and an intracerebral mechanism for the ability at birth to imitate simple articulatory movements such as opening the mouth or protusing the lips is evident from birth [Meltzoff and Moore, 1977]. Infants progressively converge toward recognizable patterns of verbal production [Kuhl and Meltzoff, 1996] and may benefit from the verbal buffer provided by the dorsal pathway to memorize and analyze speech segments [Dehaene‐Lambertz et al., 2006]. The relationship between linguistic skills at 2 years and its microstructure confirm that the arcuate is a key element during the first stages of language learning.

Premature birth is associated with a long lasting signature on whole‐brain architecture [Ball et al., 2012, 2014; Counsell et al., 2003; Nosarti et al., 2002; Salvan et al., 2014] and later neurodevelopment [Delobel‐Ayoub et al., 2009; Johnson et al., 2009; Marlow et al., 2005; Northam et al., 2012]. While the absence of a direct comparison with term control infants limits our ability to assess the full impact of premature birth, we found that increasing prematurity at birth affects arcuate fasciculus microstructure but, in the absence of severe neonatal brain injury, only minimally modulates the identified link with later linguistic skill. Although previous behavioral studies have concluded that many of the language deficits in preterm‐born children are more likely a result of general cognitive problems rather than a specific language impairment [Barre et al., 2011; Wolke and Meyer, 1999]; here, we show that the linguistic impairment in preterm born children may result from the microstructural alteration of a fundamental brain language structure.

Of importance, our results support key specific involvement of the arcuate fasciculus in language acquisition as we did not find a significant relationship with white matter microstructure in either the superior longitudinal fasciculi or the cortico‐spinal tracts. Furthermore, the identified relationship between the arcuate fasciculus and both language and cognition is in agreement with cognitive models of auditory‐verbal working memory which predict the presence of an underlying, efficient working memory buffer for language learning and processing [Baddeley, 2003; Baddeley et al., 1998]. At the age of two years, however, measures of complex linguistic skills strongly correlate to domain‐general cognitive performance. Therefore, further investigations of specific cognitive domains are needed in our subjects at an older age to distinguish measures of formal intelligence quotient, working‐memory, and attention, from phonological, syntactic processing, and semantics.

A potential limitation of this study is the use of FA as a measure of underlying white‐matter microstructure. Although we used high angular resolution diffusion‐MRI data and CSD based tractography to delineate the arcuate fasciculi, the observed relationship may be, at least in part, related to intersubject differences in the configuration of crossing fibers.

## CONCLUSION

In summary, these results validate a neurolinguistic model in which arcuate fasciculus microstructure shortly after birth plays a role in early language acquisition. We have shown that a brain‐behavior mode of covariation links linguistic performance in early childhood to a specific structure in the infant brain, which is known to support complex language function in adulthood. The microstructure of the arcuate fasciculus at around the time of normal birth underpins linguistic development at 2 years of age independent of the extreme environmental influences caused by premature extrauterine life.
